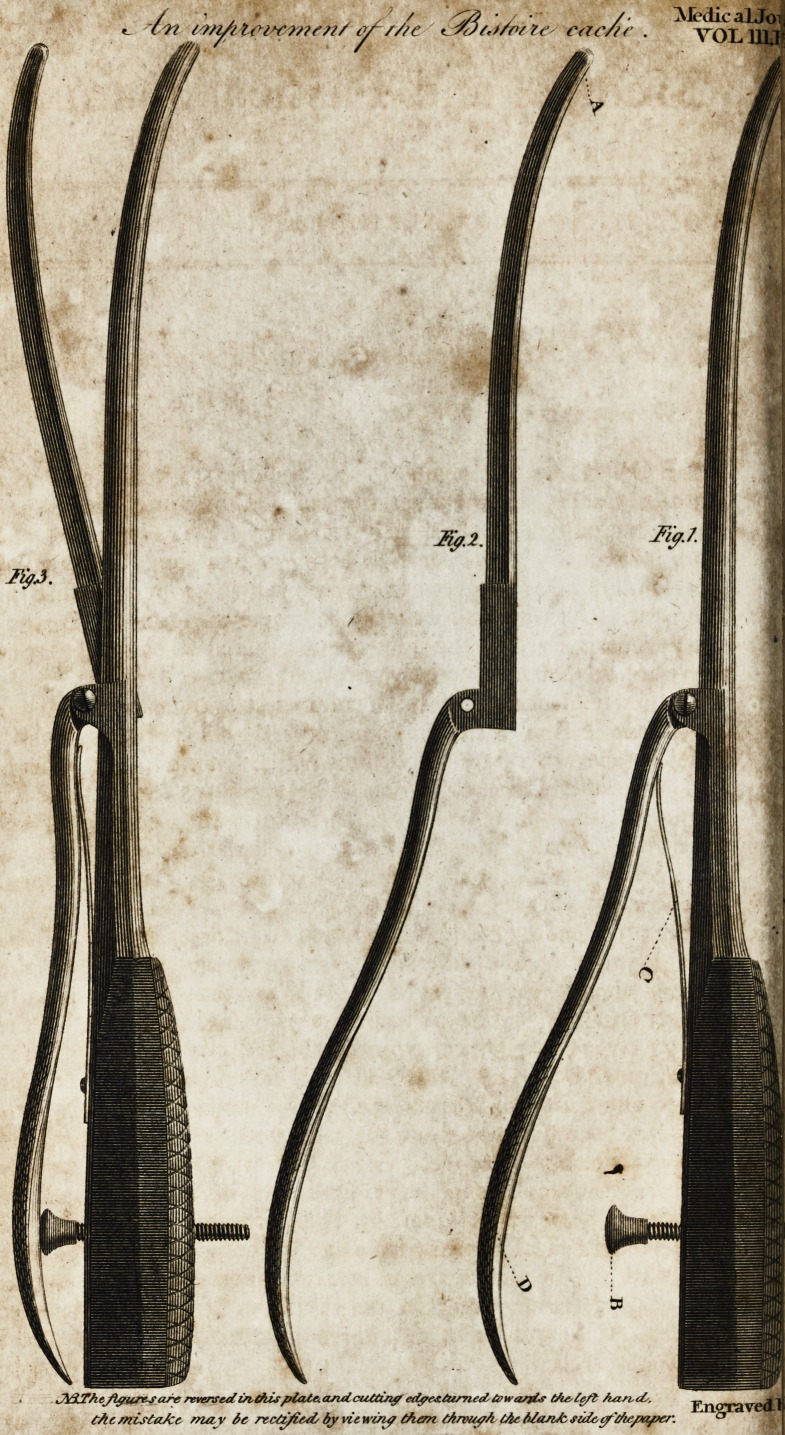# Mr. Carlisle, on the Bistoire Caché

**Published:** 1800-03

**Authors:** Anthony Carlisle

**Affiliations:** Soho-Square


### Medical and Phyfical Journal.

VOL. III.] MARCH, 1800. [NO. XIII.

### *To the Editors of the Medical and Phyfical Journal.*

GENTLEMEN,

ABOUT fix years ago I was induced to make ufe of a Biftoire Caché, in the operation of Lithotomy, fuch as had been previoufly employed in France by M. DESSAULT, and other furgeons. The operation being public, and terminating fuccefsfully, other furgeons were induced to adopt the fame inftrument. As time and experience have now feafoned this novelty in practice, and the inftrument alluded to has undergone many improvements by Mr. Stodart, an excellent workman, who, by attendance on public operations, has added a knowledge of the application of furgical apparatus to his mechanical fkill, I feel at liberty to ftate the reafons which led me into this deviation from eftablifhed cuftoms. The various alterations which eminent Lithotomifts had given to the gorget, made me fufpect the fitnefs of that inftrument for its deftination. The operations on the dead fubject ftill more encouraged my fufpicions. The conical gorgets were almoft abandoned; and the tearing effects produced by them, were found to be fufficient objections among junior practitioners. The form and extent of the cutting edge of the improved gorgets are adapted to the tafte of each operator. The obliquity of the angle, formed by the cutting end of the gorget with its barrel, has been alfo a fubject of much variety. It would be very unprofitable, at this day, to enter into a hiftory of the various modes in which Lithotomy has been performed, or the tools with which the operators have executed their work. Much experience has been already recorded, improvements have gradually been made, and we look back with thankfulnefs on thofe who have contributed to the prefervation of human life, and with hope to thofe who may ftill affift in the alleviation of mifery. It appears to me, that one very defirable point in the operation of Lithotomy, is to make a rapid and clean cut opening into the bladder. The gorget, with an edge at nearly right angles with the grooved part, is pushed through the bulbous portion of the urethra, the surrounding cellular substance, prostate gland, and whatever portion of the bladder may be cut, with a direction of force like that of digging with a spade. If the patient should be emaciated, and the surrounding cellular substance loose in its texture, the bladder may be pushed beyond the end of the staff before the tougher parts give way, and thus both apparatus slip between the bladder and rectum. I think that I have seen this happen under a very skilful hand. If the mechanical push required be considerable, the beak of the gorget is kept in the groove of the staff with difficulty and uncertainty. When a gorget with a cutting edge at a more acute angle is used, there is danger of the point reaching the furthest side of the bladder, before the shoulder gets in; or, as has really happened, it may suddenly slip, by the unforeseen yielding of the parts, and pass through the back of the bladder into the rectum. It seems convincing to my mind, that these dangers and difficulties are removed by using the Bistoire Caché. The operation being conducted in the usual manner, and the groove of the staff laid bare, this instrument is made to slide into the bladder, without either cutting or tearing. When the staff is withdrawn, the operator can measure the distance of the bladder, by feeling its posterior surface; and pulling out a little, so that his knife shall clear that surface, he executes the dark and dangerous part of this operation by a clean drawing cut. The same parts are necessarily divided in this method as when the gorget is used, and no more. The advantages of it are derived from the certainty of the opening into the bladder, and of the parts remaining in their places suffering only a simple incision.

As this account is only addressed to those who are already instructed in Anatomy and the operations of Surgery, it is not needful to add more.

Accompanying the paper is a sketch of the Bistoire Caché, in its present improved state. I am,

Gentlemen,

Your obedient servant,

*Soho-Square, Feb.* 1, 1800. ANTHONY CARLISLE.

### REFERENCE TO THE PLATE.

Fig. I. The complete instrument.

A—The wooden handle, carved, to secure it from slipping.

B—A screw, whose head is the prop of the lever D, attached to the cutting blade; and by raising or depressing this screw, the knife is made to gape a such a distance from the sheath as may suit the age, &c. of the patient.

C—A spring which presses the lever D, outwards, keeping the blade within its sheath.

D—The

D.—The lever alluded to.

Fig. II. The lever and cutting blade feparated from the handle and fheath.

A—A rounded and probe-pointed termination given to the cutting blade, for the purpofe of avoiding any end-laceration.

Fig. III. The complete inftrument, having the blade expofed, by reprefenting the lever depreffed.

## Figures and Tables

**Fig.3. Fig.2. Fig.1. f1:**